# Global research trends and hotspots of oxidative stress in diabetic retinopathy (2000-2024)

**DOI:** 10.3389/fendo.2024.1428411

**Published:** 2024-08-16

**Authors:** Kejie Du, Yichong Liu, Xintong Zhao, Haowen Wang, Xiaomei Wan, Xiaoyan Sun, Wenjuan Luo

**Affiliations:** Department of Ophthalmology, The Affiliated Hospital of Qingdao University, Qingdao, China

**Keywords:** oxidative stress, diabetic retinopathy, bibliometric analysis, NLRP3, autophagy

## Abstract

**Introduction:**

Oxidative stress has been identified as a major contributor to the pathogenesis of DR, and many diagnostic and therapeutic strategies have been developed to target oxidative stress. Our aim was to understand the contribution of the country of origin of the publication, the institution, the authors, and the collaborative relationship between them.

**Methods:**

We performed a bibliometric analysis to summarize and explore the research hotspots and trends of oxidative stress in the DR.

**Results:**

We observe an upward trend in the number of posts on related topics from year to year. Expanding on this, Queens University Belfast is the most influential research institution. Current research hotspots and trends focus on the mechanism of autophagy and NLRP3 inflammasome's role in oxidative stress in DR.

**Discussion:**

We conducted a multi-dimensional analysis of the research status of oxidative stress in diabetic retinopathy through bibliometric analysis, and proposed possible future research trends and hotspots.

## 1 Introduction

Diabetic retinopathy (DR) stands out as a paramount complication of diabetes mellitus, ranking among the primary causes of global visual impairment (1). This condition, stemming from hyperglycemia-induced microvascular complications (2), manifests through a cascade of pathophysiological events including retinal capillary dilatation, hemodynamic perturbations, tissue ischemia, and the release of diverse vasoactive substances, culminating in neovascularization (3). Furthermore, retinal neurodegenerative disorders intricately contribute to the pathomechanism of DR (4). Presently, conventional therapeutic modalities such as laser photocoagulation and vitreoretinal surgery target diabetic retinal macular edema (5) and advanced stages of DR (6), typically associated with refractory visual acuity and consequential vision-threatening side effects. Thus, there exists a pressing exigency to delve comprehensively into the underlying pathogenesis of DR, with a view to identifying novel treatment modalities for patients in the early and intermediate stages of the disease.

Oxidative stress denotes an imbalance between oxidizing agents and antioxidants within the biological milieu (7). Normally, reactive oxygen species (ROS) serve as secondary messengers in various cellular signaling pathways within the cardiovascular system (8). However, when the accumulation of ROS surpasses the antioxidant capacity, cellular oxidative stress ensues, precipitating damage to target organs and cellular injury (7). The organism’s antioxidant defense system comprises both non-enzymatic components such as vitamin C, vitamin E, melatonin, carotenoids, as well as trace elements like copper and zinc, and enzymatic counterparts including superoxide dismutase (SOD) and catalase (CAT) (9–11). The visual system, particularly the retina, is exceptionally vulnerable to oxidative stress due to prolonged light exposure, high oxygen demand associated with retinal imaging function, and the presence of easily oxidizable polyunsaturated fatty acids (PUFAs) in photoreceptors (12, 13). Oxidative stress emerges as a pivotal pathogenic mechanism in DR, precipitating structural and functional alterations in retinal vascular endothelium, mitochondrial dysfunction, apoptosis, inflammation, and lipid peroxidation. Consequently, research endeavors concerning the role of oxidative stress in DR have persisted, evolving from elucidating molecular mechanisms (14) and pathogenic effects (15) to exploring therapeutic strategies. While numerous reviews and systematic evaluations have been disseminated, it remains imperative to delineate current research focal points and prognosticate future trends, thereby establishing a robust theoretical framework for understanding DR mechanisms.

Bibliometrics examines the literature through quantitative analysis and statistics as a means of assessing the productivity and impact of relevant subject areas, as well as identifying emerging research trends. To the best of our knowledge, there have been no bibliometric studies to assess the global trends of oxidative stress in DR. In this study, we systematically analyzed oxidative stress-related studies in DR after 2000, by analyzing the most influential literature and journals in the field, keywords, key authors, major research countries and institutions, and understanding the collaborations among them, to provide an overview of the overall view of oxidative stress in DR, to summarize the current research buzz, and to predict future research trends and potential hotspots.

## 2 Materials and methods

### 2.1 Data sources and search strategies

We developed a comprehensive search strategy from Web of Science core databases, based on the MESH database and the subject term (TS). To ensure the accuracy and consistency of the searches, we completed the extraction and download of the raw data within the same day (2024.3.28). The raw data were screened by two independent researchers, and the literature that met the following requirements was included in this study:(1) the type of literature was original research and reviews (2) the language was English (3) the year of publication was after 2000, including the year 2000 (4) the literature contained complete abstracts, keywords, authors, and reference information (5) duplicated literature was excluded.

### 2.2 Statistical analysis

The meticulously curated raw data, sifted through a thorough screening process, were procured from the esteemed Web of Science platform and archived in a lucid plain text format. Our comprehensive investigation underwent rigorous statistical and visual scrutiny via the sophisticated tools of VOS viewer, Citespace, and R-Bibliometrix.

Initially, employing VOS viewer, we meticulously dissected the co-occurrence patterns of pivotal keywords, esteemed authors, and renowned institutions within the realm of published literature. Within this analysis, node dimensions were indicative of the frequency of co-occurrence, while the interconnecting lines delineated the intensity of collaboration or connection. Subsequently, utilizing Citespace, we executed a meticulous clustering of keywords and references, supplemented by a rigorous burst analysis. Subsequent to these analyses, leveraging the capabilities of R-Bibliometrix, we embarked upon a geo-visualization expedition, unraveling the geographical distribution of publications and the intricacies of inter-country collaborations. Further enriching our analytical arsenal, we harnessed additional tools such as Excel and an online mapping interface (https://ilfat-galiev.im/charticulator/) to dissect annual publication trends and the dynamics of inter-country collaborations. All threshold parameters employed in our visualization endeavors are meticulously elucidated within the figure legends, ensuring utmost transparency and replicability in our analytical pursuits.

## 3 Results

### 3.1 General overview

**Figure 1:**
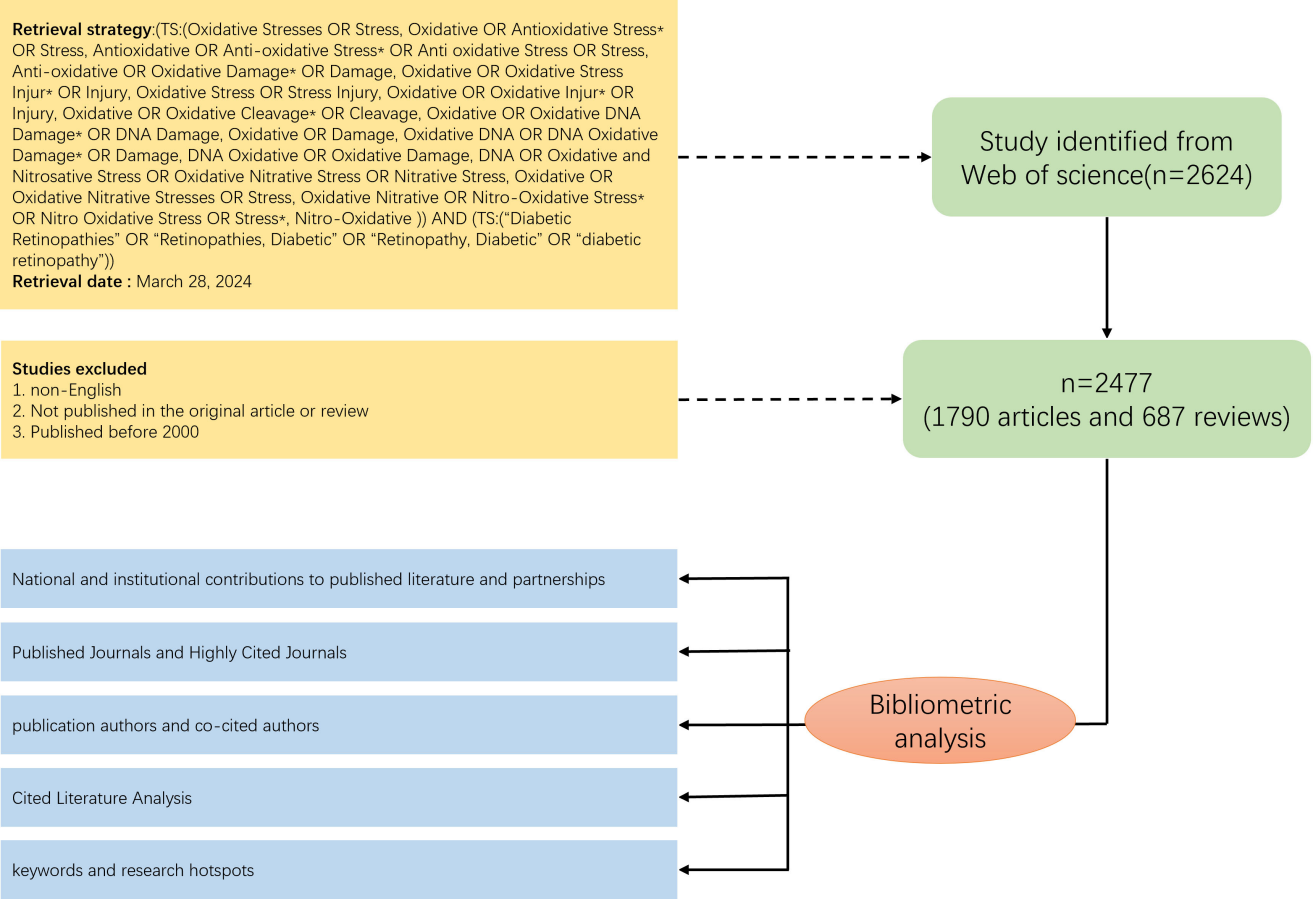
Flow chart of data collection.

**Figure 2:**
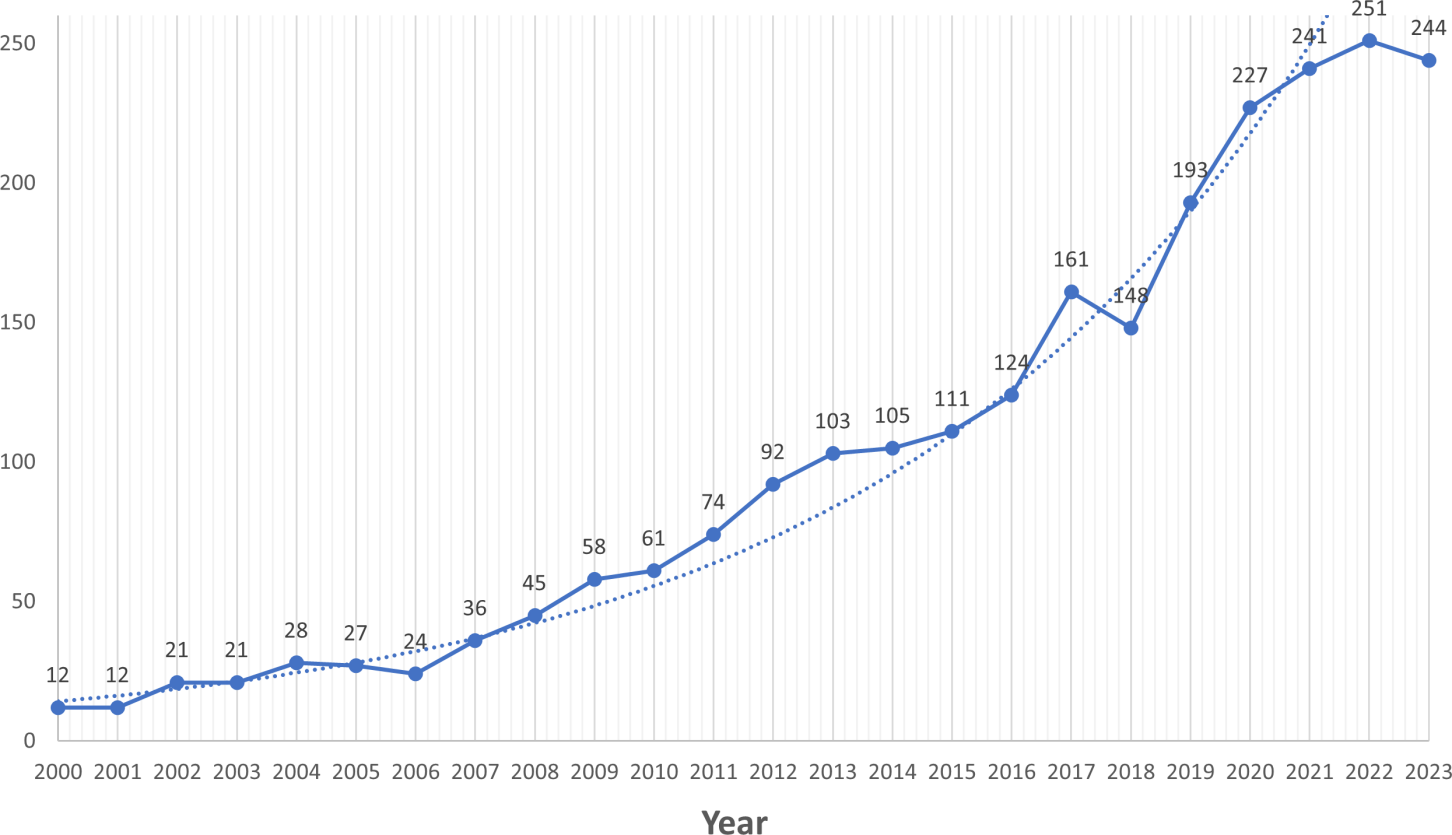
Line graph of annual publications.

According to the search strategy we developed, a total of 2624 documents were retrieved from Web of Science, and through further screening (see **Figure 1** for detailed screening process), 2477 documents, including 1790 articles and 687 reviews, were finally identified for inclusion. Overall, a total of 10,716 authors from 347 countries published articles related to oxidative stress in diabetic retinopathy in 593 journals worldwide. The total H-index of the included literature was 123, and the average number of citations for a single article was 34.91, with seven articles having more than 500 citations. Since 2000, there has been a general upward trend in the number of annual publications (**Figure 2**), with slight fluctuations and a brief decline in 2018-2019. 2022 had the highest number of publications, totaling 251.

### 3.2 National and institutional contributions to published literature and partnerships

**Figure 3:**
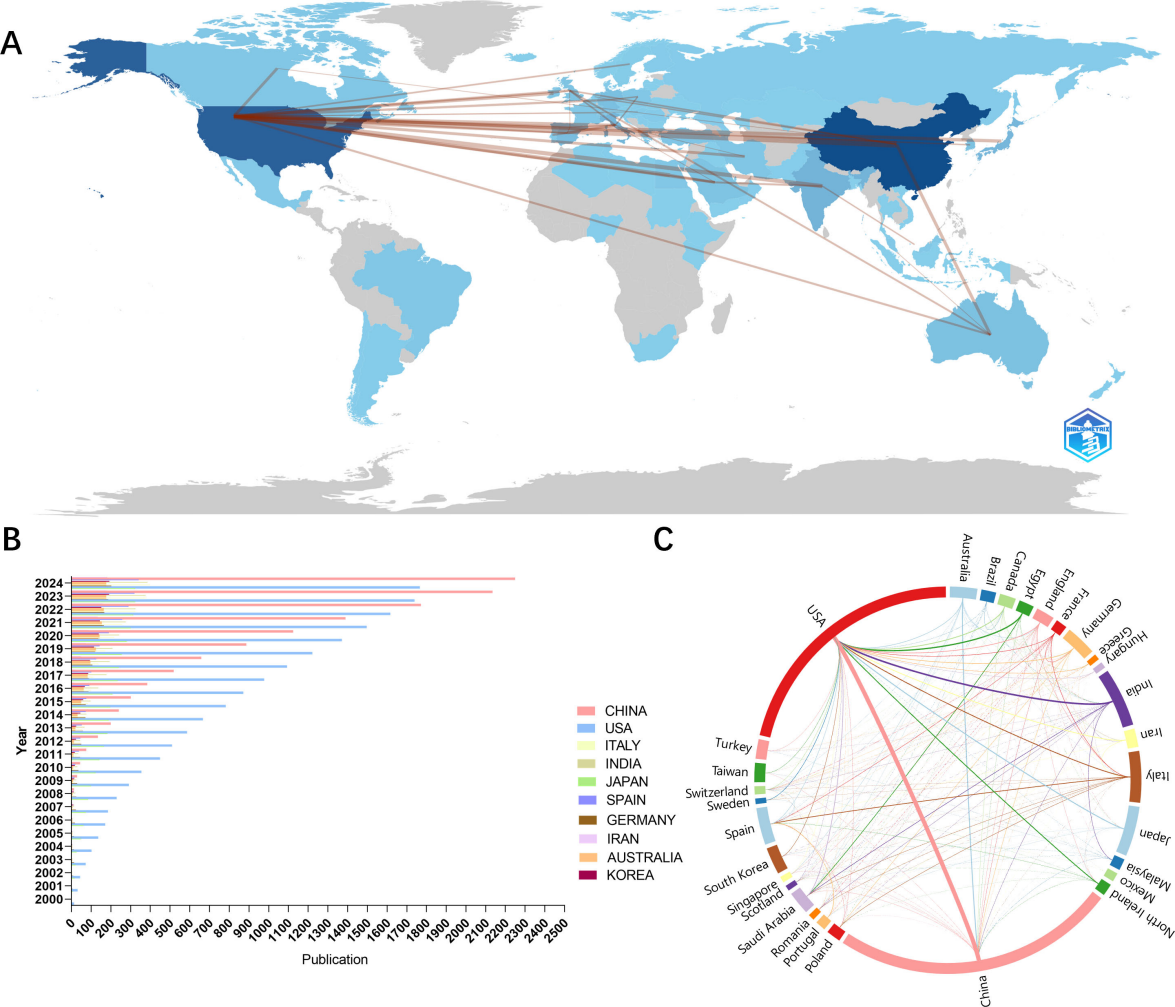
National contributions to oxidative stress in diabetic retina research. **(A)** Geo-visualization to analyze cooperation among countries **(B)** Bar chart of annual publications for the top 10 countries with the highest number of publications **(C)** Visualization of chord plots of the share of publications by country and cooperation between countries.

We analyzed the number of national publications (**Figure 3B**) and China had the highest number of publications (n=767), followed by the United States (n=682), followed by India (n=159), Italy (n=139) and Japan (n=135). In addition, the Scottish region in the UK (111.13 citations) had the highest average number of citations, followed by Singapore (96.82 citations), the Northern Ireland in the UK(84.09 citations), the United States (59.17 citations), Canada (55.89 citations) and Switzerland (54.59 citations). We found that the first countries to start studying the mechanisms of oxidative stress in diabetic retinopathy in 2000 were the United States and Japan. In the next few years, Germany, Italy, and India began to join the research team. As the concept of oxidative stress became popular and widely used, more and more countries participated in the research. In terms of international cooperation (**Figures 3A, C**), the U.S. has the closest partnership with China. At the same time, the U.S. maintains strong cooperative relationships with Egypt, India, Italy, and other countries.

**Figure 4:**
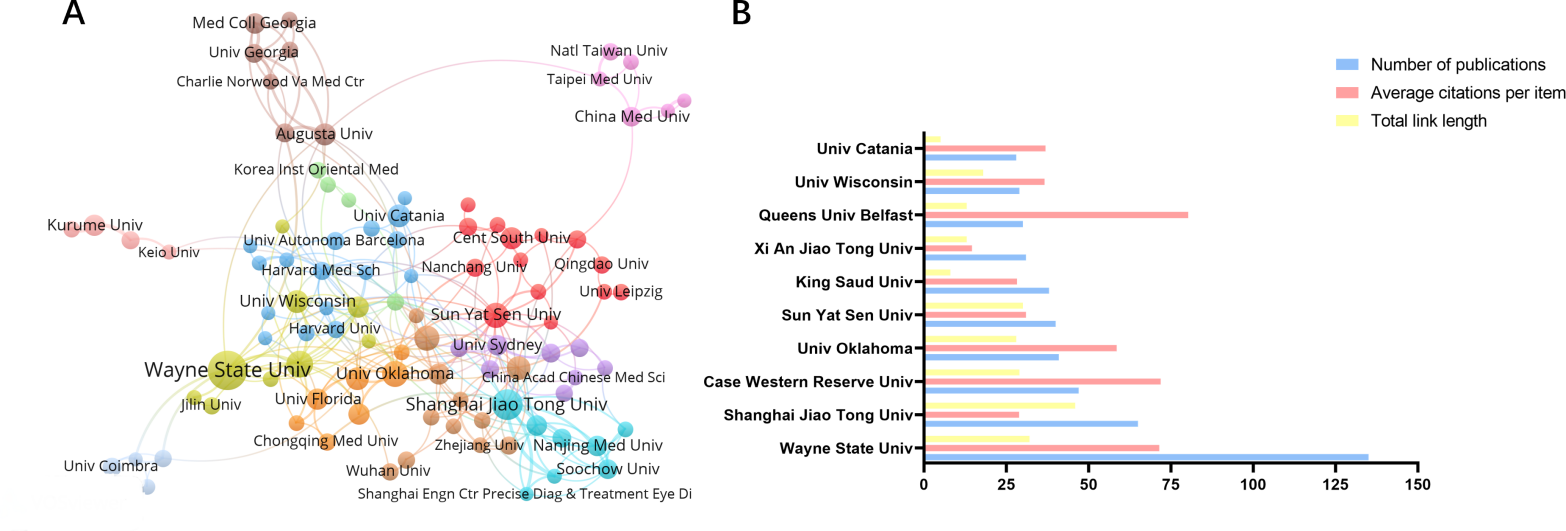
Cooperation between agencies and issuance of communications. **(A)** Collaboration between major institutions, with the threshold for institutional issuance set at 8 **(B)** Number of articles and citations for the top 10 issuing organizations.

An analysis of the highly productive institutions (**Figure 4B**) shows that the top ten institutions with the most publications are Wayne State University (n=135), Shanghai Jiao Tong University (n=65), Case Western Reserve University (n=47), Univ. Oklahoma (n=41), Sun Yat Sen University (n=40), King Saud University (n=38), Xi An Jiao Tong University (n=31), Queens University Belfast (n=30), University Wisconsin (n=29), and University Catania (n=28). The most influential institution was Queens University Belfast (Average citations per item=80.3), followed by Case Western Reserve University (Average citations per item=71.85) and Wayne State University (Average citations per item=71.45). The co-occurrence analysis of the institutions shows a relatively loose and insufficient level of collaboration between institutions. The highest impact institutions (**Figure 4A**) are currently concentrated in the United Kingdom and the United States, reflecting the fact that these two countries continue to dominate the field of research on oxidative stress mechanisms in diabetic retinopathy.

### 3.3 Analysis of published journals and highly cited journals

Since 2000, 2477 articles in the field have been published in 593 journals worldwide. The table presents the top 15 journals in the field that have published the most literature in the field (**Table 1**) and the top 15 journals with the highest number of citations (**Table 2**), ranked by frequency. The table shows that the top three journals with the most publications are “Investigative Ophthalmology & Visual Science” (n=112), “International Journal of Molecular Sciences” (n=86), and “Antioxidants” (n=76). The top three most cited journals were “Progress In Retinal and Eye Research,” “Diabetes,” and “Diabetologia”.

**Table 1:** Top 15 journals with the most publications.

journal	number of publications	if	JCR	Country
Investigative Ophthalmology & Visual Science	112	4.4	1	USA
International Journal Of Molecular Sciences	86	5.6	1	Switzerland
Antioxidants	76	7	1	Switzerland
Experimental Eye Research	53	3.4	2	USA
PLos One	52	3.7	2	USA
Diabetes	39	7.7	1	USA
Oxidative Medicine And Cellular Longevity	39	7.3	1	USA
Diabetologia	36	8.2	1	German
Frontiers In Pharmacology	34	5.6	1	Switzerland
Current Eye Research	33	2.1	3	Netherlands
Nutrients	29	5.9	1	Switzerland
Scientific Reports	29	4.6	2	England
International Journal Of Ophthalmology	28	1.4	4	China
Biomedicine & Pharmacotherapy	27	7.5	1	France
Molecular Vision	27	2.2	3	USA

**Table 2:** Top 15 most cited journals.

journal	total citations	average citation	if (2022)	JCR	Country
Progress In Retinal and Eye Research	2267	133.3529	17.8	1	England
Diabetes	4152	106.4615	7,7	1	USA
Diabetologia	3170	88.0556	8.2	1	German
Biochimica Et Biophysica Acta-Molecular Basis of Disease	728	80.8889	6.2	1	Netherlands
Diabetes-Metabolism Research and Reviews	910	75.8333	8	1	England
Free Radical Biology and Medicine	1586	75.5238	7.4	1	USA
Free Radical Research	653	72.5556	3.3	3	England
Oxidative Medicine and Cellular Longevity	2773	71.1026	7.3	1	USA
Journal of Biological Chemistry	689	68.9	4.8	2	USA
Antioxidants & Redox Signaling	712	64.7273	6.6	1	USA
Current Medicinal Chemistry	523	58.1111	4.1	2	United Arab Emirates
Investigative Ophthalmology & Visual Science	6207	55.4196	4.4	1	USA
Molecular and Cellular Biochemistry	706	54.3077	4.3	2	Netherlands
Eye	468	52	3.9	1	England
Redox Biology	563	51.1818	11.4	1	Netherlands

### 3.4 Analysis of publication authors and co-cited authors

**Figure 5:**
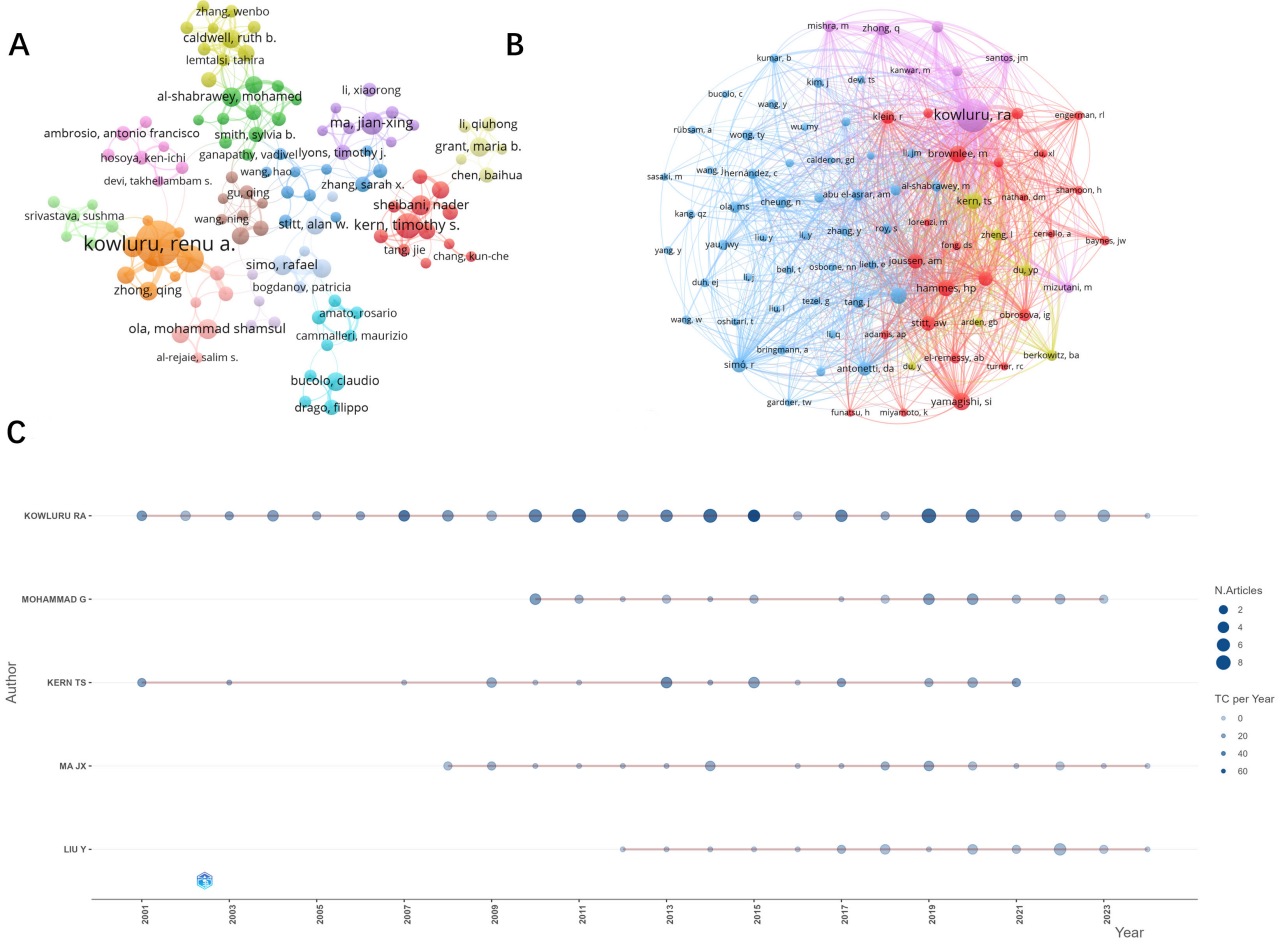
Visual analysis of publication authors and co-cited authors. **(A)** Number of publications by authors and cooperation between authors. Author posting volume threshold set at 5. **(B)** Collaboration and clustering of co-cited authors. Threshold set to 100. **(C)** Top five authors with the highest number of publications per year.

A total of 10,716 authors contributed to the included literature. The authors with the most publications in the field were, in order, Kowluru RA (n=99), Mohammad G (n=30), Kern Ts (n=28), Ma JX (n=25), and Liu Y (n=24). The results of analyzing the trend of annual publication volume of these five authors (**Figure 5C**) show that Kowluru Ra has been deeply involved in the study of oxidative stress mechanisms in DR for more than two decades. The top five most influential authors in this field are, in order, Kowluru RA (H-index=55), Kern Ts (H-index=24), Mohammad G (H-index=19), Yamagishi S (H-index=18), Mishra M (H-index=17 and) Matsui T (H-index=17). Our co-occurrence analysis of all authors (**Figure 5A**) revealed close collaboration between authors and a group approach usually centered on one influential author. For example, the most influential author, Kowluru RA, has a large collaborative network centered on him with researchers such as Manish Mishra, Mohammad, Ghulam, Zhong, qingyang, Dos Santos, Julia Matzenbacher, and others.

The co-occurrence analysis network of the cited authors (**Figure 5B**) presents four clusters, and the authors in the figure are highly influential in the research field of oxidative stress mechanisms in diabetic retinopathy. The top three most influential authors according to the visualization analysis are, in order, Kowluru, Ra (citations = 2720), Yamagishi, Si (citations = 621) and Brownlee, M (citations = 570).

### 3.5 Cited literature analysis

In order to analyze the citation trends in recent years, we quantitatively analyzed the cited literature. The top 15 most cited papers in the field (**Table 3**) are presented in the table and ranked according to the number of citations. As can be seen from the table, the top two most cited publications are “Oxidative Stress and Diabetic Complications” by Ferdinando Giacco et al., 2010 (16) published in “Circulation Research” and “Epidemiology of diabetic retinopathy, diabetic macular edema and related vision loss” by Ryan Lee et al.2015 (17) published in “ Eye and Vision”. These two review articles have largely set the tone for the study of oxidative stress mechanisms in diabetic retinopathy.

**Table 3:** Top 15 most cited literature.

Title	Published year	First author	journal	Total citations	average citation per year
Oxidative Stress and Diabetic Complications	2010	Ferdinando Giacco	Circulation Research	3562	254.43
Epidemiology of diabetic retinopathy, diabetic macular edema and related vision loss	2015	Ryan Lee	Eye and Vision	877	97.44
The Role of the Reactive Oxygen Species and Oxidative Stress in the Pathomechanism of the Age-Related Ocular Diseases and Other Pathologies of the Anterior and Posterior Eye Segments in Adults	2016	Małgorzata Nita	Oxidative Medicine and Cellular Longevity	776	97.00
Diabetic Retinopathy and Diabetic Macular Edema: Pathophysiology, screening, and novel therapies	2003	Thomas A. Ciulla	Diabetes Care	712	33.90
Diabetic retinopathy: current understanding, mechanisms, and treatment strategies	2017	Elia J. Duh	JCI Insight	572	81.71
Use of aminoguanidine (Pimagedine) to prevent the formation of advanced glycation endproducts	2003	Paul J Thornalley	Archives of Biochemistry and Biophysics	510	24.29
Para-inflammation in the aging retina	2009	Heping Xu	Progress in Retinal and Eye Research	505	33.67
Oxidative Stress and Diabetic Retinopathy	2007	Renu A. Kowluru	Journal of Diabetes Research	482	28.35
Role of Inflammation in Diabetic Retinopathy	2018	Anne Rübsam	International Journal of Molecular Science	437	72.83
Reactive oxygen species as mediators of angiogenesis signaling. Role of NAD(P)H oxidase	2004	Masuko Ushio-Fukai	Molecular and Cellular Biochemistry	372	18.60
Abnormalities of Retinal Metabolism in Diabetes and Experimental Galactosemia: VII. Effect of Long-Term Administration of Antioxidants on the Development of Retinopathy	2001	Renu A. Kowluru	Diabetes	366	15.91
Diabetic Macular Edema: Pathophysiology and Novel Therapeutic Targets	2015	Arup Das	Ophthalmology	348	38.67
Oxidative stress and diabetic retinopathy: Molecular mechanisms, pathogenetic role and therapeutic implications	2020	Qingzheng Kang	Redox Biology	347	86.75
Glycation and Carboxymethyllysine Levels in Skin Collagen Predict the Risk of Future 10-Year Progression of Diabetic Retinopathy and Nephropathy in the Diabetes Control and Complications Trial and Epidemiology of Diabetes Interventions and Complications Participants With Type 1 Diabetes	2005	Saul Genuth	Diabetes	338	17.79
Protein Glycation A Firm Link to Endothelial Cell Dysfunction	2004	Jean-Luc Wautier	Circulation Research	335	16.75

**Figure 6:**
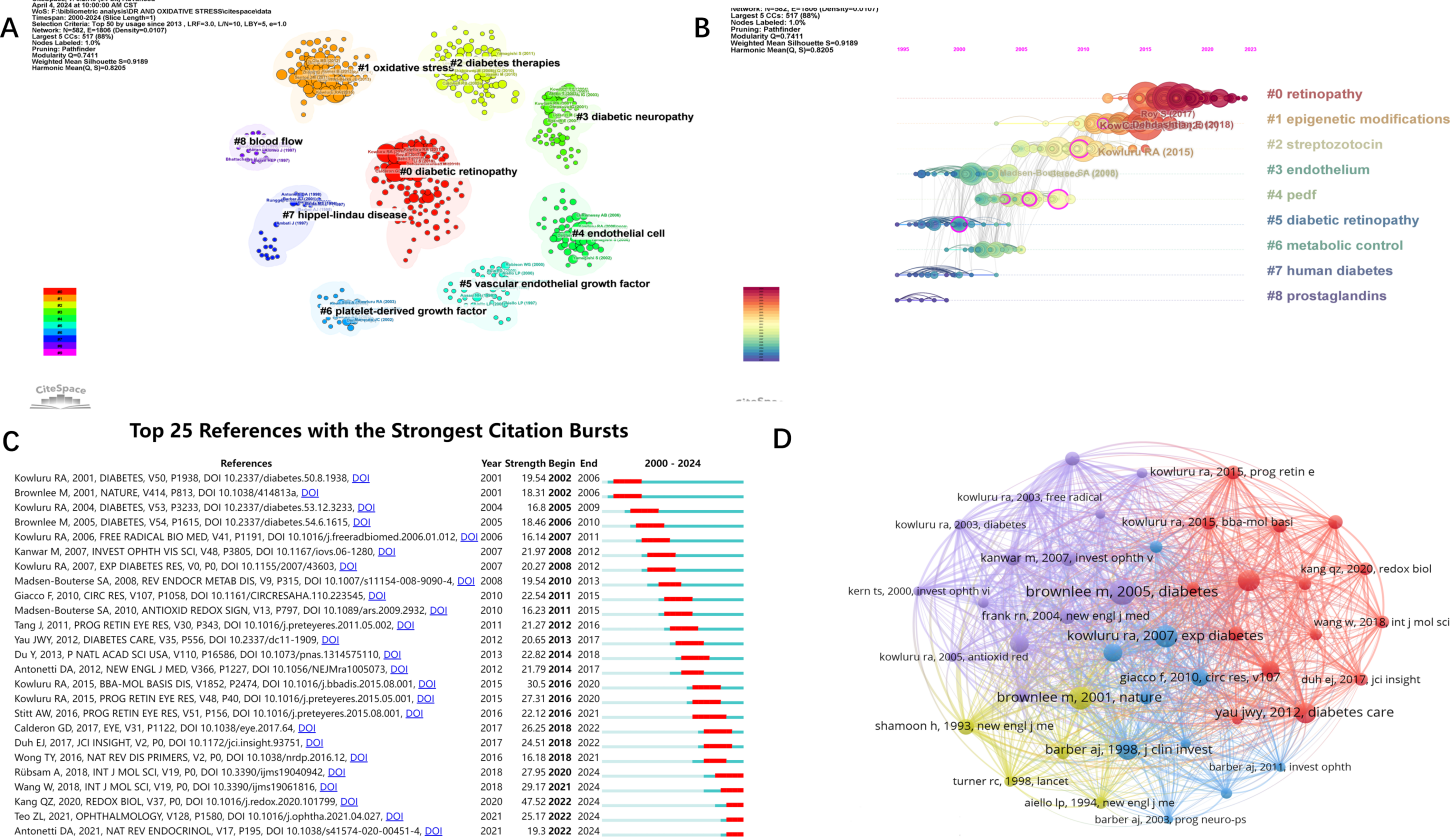
Visual analysis of references. **(A)** Clustering references by keywords. **(B)** Timeline analysis of the results of reference clustering. **(C)** Burst analysis of references. **(D)** Network visualization analysis and clustering of co-cited literature. Threshold set to 80.

We performed a keyword-based cluster analysis of the cited literature (**Figure 6A**), and the leading cluster labels were retinopathy, epigenetic modification, streptozotocin, endothelial, and pigment epithelium-derived factor (PEDF). Time-zone analysis of the clusters (**Figure 6B**) showed that the highest level of interest in the last few years was in epigenetic modification of retinopathy. In addition, we performed burst analysis on the references (**Figure 6C**). Burst analysis refers to a steep increase in the number of references at a specific time, and the red line indicates the time of the burst. The results showed that five references showed a burst of citations in the recent past, namely “Role of Inflammation in Diabetic Retinopathy” published by Anne Rübsam et al. in 2018 (18), “Diabetic Retinopathy: Pathophysiology and Treatments” published by Wei Wang et al. in 2018 (2), “ Oxidative stress and diabetic retinopathy: Molecular mechanisms, pathogenetic role and therapeutic implications” published by QingZheng Kang et al. in 2020 (3), “Global Prevalence of Diabetic Retinopathy and Projection of Burden through 2045” published by ZhenLing Teo et al. in 2021 (19), and “Current understanding of the molecular and cellular pathology of diabetic retinopathy” published by David A. Antonetti et al. in 2021 (20). Among them, the article by JingZheng Kang et al. has the highest outbreak value (47.52).

The **Figure 6D** shows the co-occurrence analysis and network visualization of co-cited literature. A reference is defined as a co-cited literature if it is cited by multiple articles in the field. The top three co-cited literature were Brownlee m et al., Diabetes, in 2005 (co-cited 254 times), Brownlee m et al., Nature, in 2001 (co-cited 224 times) and kowluru RA et al., Experimental Diabetes Research, in 2007 (co-cited 220 times).

### 3.6 Analysis of keywords and research hotspots

**Figure 7:**
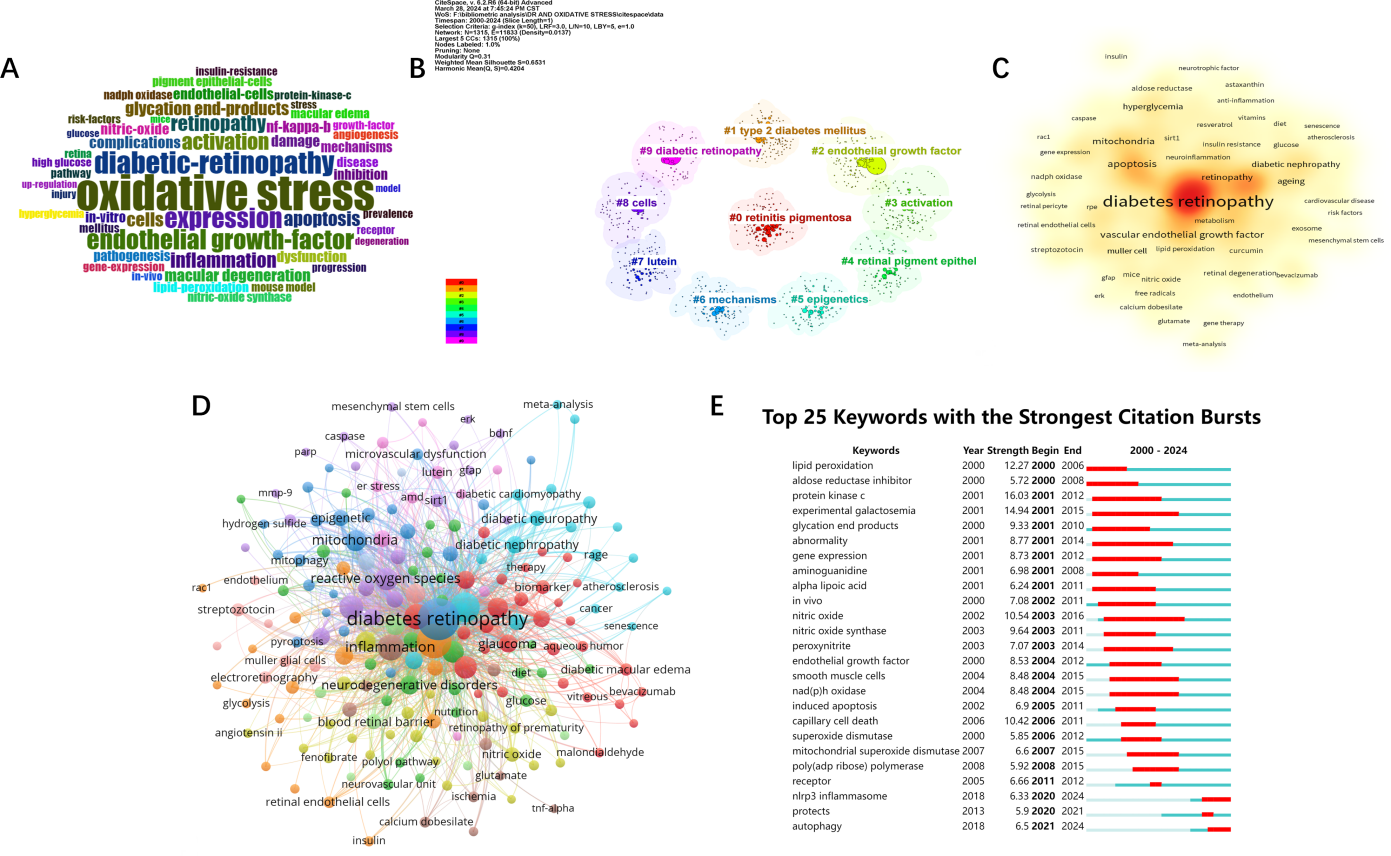
Visual analysis of keywords and research hotspots. **(A)** word cloud of keywords. **(B)** Cluster analysis of keywords. **(C)** Keywords Heatmap. **(D)** Keyword network analysis. Threshold set to 6. **(E)** Burst analysis of keywords.

Author keywords in the literature often summarize the topic and direction of the article, so analyzing keywords is helpful for us to understand and distinguish different research topics and explore emerging research hotspots and trends. Firstly, a word cloud(**Figure 7A**) was generated by R-bibliometrix to visualize the high-frequency keywords. Then, among the 3,922 keywords obtained from VOSviewer screening, a total of 219 keywords appeared more than 6 times. We merge the repeated or similar keywords among them, and then perform co-occurrence analysis(**Figure 7B**). The node size indicates the frequency of occurrence, and the connecting line between the nodes represents the strength of the connection between the keywords. The visualization of the heat analysis (**Figure 7C**) was also done, and it was found that in addition to “DR” and “oxidative stress”, “inflammation”, “metabolism “, “apoptosis”, “vascular endothelial growth factor”, “mitochondria” and “antioxidants”, in addition to “DR” and “oxidative stress”. “ etc. appear with high frequency and are popular keywords. In order to have a more in-depth understanding of the keywords, we used citespace to perform cluster analysis (**Figure 7D**) and burst analysis of the keywords(**Figure 7E**). The clustering of the top ten keywords was visualized in the order of “retinitis pigmentosa”, “type 2 diabetes”, “endothelial growth factor”, “ activation”, “retinal pigment epithelium”, “epigenetics”, “mechanism”, “lutein “, “cellular”, and “DR” tags were clustered. According to the results of the burst analysis, the keywords that have been exploding recently are “autophagy” and “NLRP3 inflammatory vesicles”. As mentioned above, the study of inflammation, apoptosis, vascular endothelial growth factor, and mitochondria has been the main research direction in the study of oxidative stress mechanism in diabetic retinopathy. And autophagy and NLRP3 inflammatory vesicles are likely to be the hotspot and research trend at present and in the coming time.

## 4 Discussion

In this study, we conducted a systematic bibliometric analysis of oxidative stress in the field of diabetic retinopathy from 2000-2024. To the best of our knowledge, this should be the first in-depth bibliometric analysis of oxidative stress in the field of diabetic retinopathy, which helps to understand the current state of research and to grasp the research trends and directions in the field. The results of the analysis showed a significant and steady upward trend in the number of annual publications since 2000, except for a slight fluctuating decline in 2018, with the highest number of publications in 2022. These results indicate that this field of research has a great potential for research and a positive future with an increasing number of researchers involved.

Among all the journals globally, the largest number of publications is concentrated in the United States and Switzerland. Investigative Ophthalmology & Visual Science from USA, International Journal Of Molecular Sciences from Switzerland and Antioxidants are the top three contributing journals. The type of journals shows that most of the articles in the field are published in Ophthalmology, Endocrinology and Biochemistry & Molecular Biology journals, while others are published in general interest journals. In terms of citations, the most influential journal affiliations were centered in the United States, the United Kingdom, and the Netherlands. Progress In Retinal and Eye Research in the UK, Diabetes in the US and Diabetologia in Germany were the most cited journals. In addition, Diabetes, Oxidative Medicine and Cellular Longevity and Diabetologia appeared in the Top 15 of both highly published and highly cited journals. Among all the articles included, China and the United States accounted for the largest proportion of articles, accounting for 58.58% of the total number of articles published. This may stem from the rich financial and resource support from the country. Wayne State University in the U.S. is the institution with the highest number of articles, followed by Shanghai Jiao Tong University in China. Four of the top ten institutions are located in the United States and three in China. It is also worth noting that Queen’s University of Belfast, although it does not have as many publications as the previous institutions in China and the United States, has the most citations and is also a very influential and promising institution.

When viewed through the lens of authorship, Kowluru RA emerges as an eminent figure, significantly contributing to and influencing the domain of diabetic retinopathy. Hailing from the Department of Ophthalmology, Vision, and Anatomical Sciences at Wayne State University, Kowluru RA has dedicated over two decades to unraveling oxidative mechanisms within this field. With a prolific publication record and top-ranking co-citations, he has cultivated a comprehensive and multifaceted research portfolio, delving into topics spanning mitochondria, non-coding RNAs, epigenetic modifications, oxidative enzymes, and homocysteine.

Within our examination of the literature, we have distilled the top 15 highly cited articles. Among these, 13 are reviews, while 2 are experimental studies. Ferdinando Giacco et al. (2010) (16) stands out as the most cited work, elucidating the pivotal role of metabolite-generated reactive oxygen species (ROS) in diabetic complications. Highlighting five mechanisms whereby mitochondrial superoxide inhibits the glycolytic enzyme NADPH, this seminal piece underscores the profound impact of oxidative stress in diabetes pathogenesis. Masuko Ushio-Fukai et al. (2004) (21) proposed ROS as crucial mediators of angiogenesis and vascular disease risk factors. Their exploration of NADPH oxidase-induced ROS production in endothelial cells unveils a potential mechanistic link to angiogenic phenotypes in diabetic retinopathy, paving the way for future investigations. In their seminal work, Kowluru RA et al. (2001) (22) evaluated antioxidant supplementation’s effects on diabetic retinopathy development using diabetic and galactosemic mouse models. Demonstrating the efficacy of long-term antioxidant administration in mitigating early disease progression, their findings advocate for antioxidant therapy’s integration into diabetic retinopathy management. Subsequent contributions by Thomas A. Ciulla et al. (2003) (23), Elia J. Du et al. (2017) (24), and others elucidate the pathophysiological underpinnings, screening modalities, and therapeutic interventions for diabetic retinopathy. Bridging basic scientific insights with clinical practice, these endeavors enrich diagnostic and therapeutic approaches. Qingzheng Kang et al. (2020) (3) offer a contemporary update on oxidative stress mechanisms, therapeutic strategies, and potential targets for antioxidants in diabetic retinopathy. Their comprehensive review encompasses plant-derived antioxidants and chemical-pharmacological agents such as quercetin, curcumin, and resveratrol, underscoring their potential in oxidative stress management.

To attain a more nuanced comprehension of the domain and to delve into its research focal points and trajectories, we undertook a co-occurrence analysis of pivotal keywords, sieving out those exhibiting heightened frequency. Foremost among these were “diabetic retinopathy” and “oxidative stress”, whilst “inflammation”, “apoptosis”, “antioxidants”, “mitochondria”, and “angiogenesis” also manifested as recurrent motifs. The investigation of the inflammatory response and cellular demise induced by hyperglycemia in diabetic retinopathy emerged as central research themes, branching into an exploration of associated pathways, cytokines, and phenotypes. For instance, Hui Kong et al. (2022) (25) scrutinized the *in vivo* and *in vitro* anti-inflammatory properties of 3TC in a murine model of diabetic retinopathy, probing the impact of targeting the P2X7/NLRP3 signaling cascade on apoptosis and pyroptosis. This line of research investigating the effects of molecular agents or substances on phenotypic outcomes through signaling cascades is currently the dominant research paradigm in *in vitro* experiments.

Following the empirical validation of the beneficial effects of antioxidant supplementation on the onset of early diabetic retinopathy by Kowluru RA et al. (22), endeavors focusing on antioxidants for diabetic retinopathy management have proliferated, yielding a plethora of novel antioxidant agents and therapeutic modalities (26). In recent years, melatonin, an endogenous anti-inflammatory and antioxidant agent, has garnered considerable scientific interest owing to its potent pharmacological attributes. A multitude of studies have evinced melatonin’s capacity to counteract the hyperglycemia-induced upsurge in oxidative stress biomarkers such as superoxide dismutase (SOD) and glutathione-s transferase (GSH). Furthermore, melatonin (27) has demonstrated a suppressive effect on the expression of inflammatory mediators including tumor necrosis factor-alpha (TNF-alpha) and interleukin-6 (IL-6), thereby exerting robust anti-inflammatory effects. Moreover, melatonin has exhibited ameliorative effects on neurodegenerative cerebral lesions (28).

In their work, Oke-Oghene Philomena Akpoveso et al. (2023) (29) delineated the prospective utility of prominent plant-derived antioxidants in mitigating diabetic complications. The study posited that polyphenols such as caffeic acid phenethyl ester (CAPE) and resveratrol could safeguard mitochondrial membrane potential and impede necroptosis and apoptosis by modulating mitochondrial ion channels. Additionally, it was proposed that these compounds might augment GPx4 and coenzyme Q (CoQ) levels, thereby inhibiting lipid peroxidation and ferroptosis (30–32). Intravitreal administration of anti-VEGF agents currently stands as the frontline therapeutic intervention for diabetic retinopathy, effectively mitigating diabetic macular edema. Notably, recent investigations have spotlighted the inhibitory effect of VEGF-B on retinal apoptosis, with promising implications for clinical translation, as corroborated by experimental and clinical evidence (33).

Through burst analysis, two prominent keywords, “autophagy” and “NLRP3 inflammasome,” emerge as current and future focal points in research. Chang et al. (2022) (34) comprehensively delineated the intricate interplay between autophagy and oxidative stress. They elucidated how reactive oxygen species (ROS), stemming from oxidative stress, stimulate transcription factors to orchestrate the expression of autophagy-related genes. Furthermore, ROS induction of autophagy occurs via signaling cascades such as AMPK/mTORC1 and PI3K/AKT pathways. Despite ROS-induced autophagy, feedback mechanisms exist to modulate ROS levels through processes like mitochondrial autophagy, thus preserving redox homeostasis. Notably, in diabetic retinopathy (DR), autophagy curtails inflammatory vesicle activation and safeguards retinal endothelial cells. However, excessive autophagy precipitates pericyte demise, exacerbating disease progression. Current *in vitro* investigations spotlight autophagy’s diverse roles in DR pathogenesis across retinal cell types including astrocytes, ganglion cells, microglia, and pericytes (35, 36). Bioinformatics analyses (37) have identified potential autophagy-related genes in DR. Concurrently, Feng et al. (2022) (38) probed the impact of the autophagy-lysosomal pathway in retinal pigment epithelial cells, while Liu et al. (2022) (39) demonstrated the inhibitory effect of glial maturation factor-β (GMFB) on this pathway, elucidating its implications in iron-induced apoptosis. Chaurasia et al. (2018) (40) illustrated in the Akimba type 1 diabetic mouse model how NLRP3 inflammasomes impair retinal immune function and foster neoangiogenesis in late-stage DR. Recent investigations have centered on modulating ROS/NLRP3 signaling pathways, shedding light on apoptosis, pyroptosis, and potential therapeutic targets in DR (25, 41). Additionally, Zheng et al. (2023) (42) discerned through transcriptomic analysis of mouse retina that IncRNA (181-Rik) mitigates ischemia-reperfusion injury (IR) by quelling NLRP3 inflammatory pathway activation. Beyond DR (43, 44), NLRP3 assumes pivotal roles in various ocular inflammatory diseases, underscoring its vast research prospects.

This study also has some limitations. The articles included in this study were all from Web of Science and there may be some articles that were not included. While some articles may be overlooked, the database’s repository of high-quality research ensures the fidelity of the overall research trajectory.

## 5 Conclusion

As the exploration of oxidative stress in diabetic retinopathy (DR) continues, it becomes imperative to delve into its historical trajectory, scrutinize existing findings, and anticipate future research trajectories. Remarkably, the realm of bibliometric analysis remains uncharted in this domain, a void which our present study endeavors to fill. Our analytical findings unveil a steady escalation in research fervor surrounding oxidative stress in DR over the past two decades. This temporal evolution has witnessed a dynamic shift in research focus, unveiling emergent thematic frontiers. Notably, autophagy and the role of NLRP3 inflammatory vesicles emerge as paramount arenas warranting scholarly exploration. We posit that the quantitative insights gleaned from our study hold the key to comprehending the nuanced ebbs and flows within this scholarly domain. Furthermore, they furnish invaluable guidance and fertile ground for future inquiry, equipping researchers with nuanced perspectives and research trajectories.

## Data Availability

The original contributions presented in the study are included in the article/supplementary material. Further inquiries can be directed to the corresponding author.
